# Reports of Meetings

**Published:** 1952-01

**Authors:** 


### Reports of Meetings

### BRISTOL MEDICO-CHIRURGICAL SOCIETY

The Annual General Meeting of the Society was held in the University at 8.15 p.m. on Wednesday, October 10th, 1951. The President, Mr. A. W. Jackman, resigned the chair to Dr. Victoria Tryon, President for 1951–52, who then gave her Presidential address entitled, **" Women in Medicine "**.

*Officers for* 1951

*President*, Dr. Victoria Tryon. *President-Elect*, Mr. E. Watson-Williams. *Hon. Secretary*, Mr. A. L. Eyre-Brook. *Hon. Treasurer*, Dr. G. E. F. Sutton. *Hon. Medical Librarian*, Professor A. Rendle Short. *University Library Committee Representatives*, Dr. S. F. Marwood, Mr. H. Keith Lucas.

*Committee:* Dr. W. Bain, Dr. Beryl Corner, Dr. W. H. Hilton, Dr. A. B. Kettle, Dr. J. E. Lucas, Dr. A. M. Maclachlan, Dr. S. F. Marwood, Mr. F. D. Murphy, Dr. S. Silvey.

*Editor of the Journal*, Mr. H. Keith Lucas. *Assistant Editor*, Dr. J. E. Cates.

### CLINICAL MEETING OF THE BRISTOL MEDICO-CHIRURGICAL SOCIETY

*Frenchay Hospital, 14th November,* 1951.

*The following report has been received from our roving correspondent.*

I found the clinical meeting at Frenchay Hospital very interesting and certainly very well arranged. Even before I reached the demonstrations, I was filled with admiration for the well-organized parking arrangements and for the clear directions guiding those who do not know their way about the labyrinths of Frenchay Hospital.

As always, at clinical meetings, I found I spent too much time in the first few groups of specimens and cases, and had to hurry a little through the later ones. One reason for this is that, as a general practitioner, I find myself more concerned with the early history of a case than with the later, more specialist management. This usually entails considerable discussion with the patient, and cannot be hurried. This time-consuming factor was less marked at the Frenchay meeting than is usual, as the case notes and records were excellently displayed, particularly in the cases presented by the Neuro-surgical unit. The importance of photographs as case records was well shown by the cases demonstrated by the Plastic unit: I would have liked to see one completed case for I still find it difficult to visualize the end result, particularly where the cosmetic result is important.

The refreshments provided were as admirable as one has come to expect in a hospital. The discussion afterwards was not so good, and came as an anti-climax; I later regretted that the time could not have been used in further study of the cases. Except in the very interesting case of chordotomy and ureteric transplant to the caecum for pelvic carcinoma, which admittedly could not easily be discussed in front of the patient, the brief " lectures " given were, I thought, largely redundant. The warning concerning the ease with which bacteria become resistant to antibiotics was timely and thought-provoking. A very interesting discussion could have been developed if more detailed advice had been available on the optimum dosage of the different preparations of penicillin.

These minor points for criticism did not in any way detract from a very stimulating and enjoyable evening which made me regret that such meetings cannot be held more often. Our grateful thanks are due to the staff of Frenchay and Cossham Hospitals, and to the officers of the Society, for their careful organization.

### DEVON AND EXETER MEDICO-CHIRURGICAL SOCIETY

At a meeting on 15th November, 1951, **Dr. N. S. Alcock** spoke of the diagnostic value of Electroencephalography. Berger in 1929 found that the normal brain produced rhythmic waves demonstrable by means of the electroencephalogram. Later, Lennox found that disturbances in the normal wave-rhythm occured during epileptic fits and that the graphs produced by petit and grand mal differed markedly from each other. Thus, petit mal produced a *wave-and-spike* which continued throughout the attack. Grand mal, on the other hand, produced a graph showing a series of spikes of fairly constant periodicity, about twenty-five to the second. Further it was found that by subjecting the epileptic patient to various stimuli fits could be induced. One such stimulus, harmless to the patient was to subject him to flashes of light at the rate of about eighteen per second. By spacing electrodes at intervals over the head electrical disturbances at the site of brain lesions are set up so that such lesions can be localized with considerable accuracy between the two electrodes chiefly affected. Further, from the nature of the waves, evidence can be obtained as to whether the lesion is inflammatory or neoplastic in nature.

### SECOND INTERNATIONAL POLIOMYELITIS CONFERENCE, SEPTEMBER 3RD TO 7TH, 1951

In Copenhagen the poliomyelitis conference was a medical and social education. Arranged very largely by America it had an organisational smoothness which left one breathless and yet predominant memories are of noise—flash-bulbs, movie and television apparatus, loud-speakers, interpreters, and talk.

It seemed that too large a proportion of the time was devoted to intricate virological and immunological studies, and often clinical medicine with its cases of poliomyelitis was a far cry from monkeys, suckling baby mice and logarithmic graphs. Yet the bubbles on the pot indicated that advances are being made.

Dr. Enders of Boston has grown the Brunhilde, Lansing and Leon strains of poliomyelitis virus in tissue culture. He has subcultured and titrated the virus and his work bids fair to eliminate the use of the expensive monkey, and, since there is evidence of diminished virulence on tissue subculture, the possibility of an effective vaccine becomes a little nearer.

Dr. Casals of New York reported on what appears to be a reliable complement fixation test for poliomyelitis. This, if capable of general application, might end the doubt in non-paralytic cases and make epidemiological work swifter and surer.

It was useful to meet so many people all doing something to control the disease, and it was good to know that we are not missing anything of real importance to the welfare of our patients. All the same, a commercial exhibition of mechanical respirators made one think ill of the Nuffield iron lung which has now worn itself out doing good deeds in this country.

The hospitality of the Danes is something to experience rather than to hear about. The conference must have required infinite pains from all the organisers, but especially the work of Dr. H. C. A. Lassen cannot easily be forgotten.

